# Quantifying microstructural changes in retinitis pigmentosa using spectral domain – optical coherence tomography

**DOI:** 10.1186/s40662-019-0139-0

**Published:** 2019-05-15

**Authors:** B. Poornachandra, Aruj K. Khurana, Preethi Sridharan, Priyansha Chatterjee, Chaitra Jayadev, Naresh Kumar Yadav, Rohit Shetty

**Affiliations:** 1 Department of Vitreo Retina, Narayana Nethralaya Eye Institute, Bangalore, India; 2 Department of Ophthalmology, Narayana Nethralaya Eye Institute, 121/C, Chord Road, 1 “R” Block, Rajajinagar, Bangalore, Karnataka 560010 India

**Keywords:** Retinitis Pigmentosa, Photoreceptor outer segment length (PROS), Foveal outer segment pigment epithelial thickness (FOSPET), Ellipsoid zone (EZ), FOSPET-PROS ratio (FPR)

## Abstract

**Background:**

Most patients of established retinitis pigmentosa (RP) have subnormal peripheral vision and heavily rely on central vision for their daily activities. Central visual acuity is dependent on photoreceptor survival at the macula. Identification of structural changes that precede visual loss is essential. The aim of this study was to correlate the Spectral Domain-Optical Coherence Tomography (SD-OCT) characteristics with visual acuity in patients with typical RP.

**Methods:**

This was a retrospective, observational case series of 224 eyes of 113 RP patients conducted a tertiary eye care center. SD-OCT imaging was done for all eyes. Central retinal thickness (CRT), photoreceptor outer segment length (PROS), foveal outer segment pigment epithelial thickness (FOSPET) and ellipsoid zone (EZ) extent were measured. A new variable, FOSPET-PROS ratio (FPR), obtained by dividing FOSPET by PROS is defined and correlated to corrected distance visual acuity (CDVA) in logMAR using linear regression.

**Results:**

Out of 113 patients, 71 were males and 42 females. Mean age of the patients was 35.4 ± 15.1 years. Mean CDVA was 0.33 ± 0.25 logMAR with no difference between the genders. Mean CRT (218.74 ± 83.5 μm) and FPR (1.63 ± 0.22) significantly correlated to CDVA with a correlation coefficient of r = − 0.139 (*p* = 0.048) and r = 0.842 (*p* = 0.0001), respectively. FOSPET (mean = 71.15 ± 13.8 μm) and PROS (mean = 44.85 ± 12.5 μm) did not show a significant correlation to CDVA, independent of FPR.

**Conclusions:**

Retinal microstructural changes on SD-OCT, especially the FPR, can be used as a surrogate marker to monitor disease progression in the central retina in degenerative diseases like RP.

## Background

Retinitis pigmentosa (RP) is a complex hereditary disease characterized by progressive degeneration of photoreceptors [1]. Worldwide prevalence of RP ranges from 1:1878 to 1:7000 across different racial groups [1]. Prevalence in India has been reported to be much higher, ranging from 1:372 in rural to 1:950 in urban populations [2]. Mutations in multiple genes, many of which encode proteins that are essential for photoreceptor structure and function are said to be the cause for RP [3]. The key fundus features include attenuation of retinal arterioles, ‘waxy pallor’ of the optic nerve head and ‘bone-spicule’ retinal pigment, thinning and atrophy of the retinal pigment epithelium in the mid- and far-peripheral retina and characteristic electroretinogram findings of diffuse photoreceptor disease [1].

Patients typically present with visual impairment involving night and peripheral vision with gradual deterioration of the central visual acuity. Photoreceptor survival in central retina correlates closely with visual function in these patients [4]. Therefore, assessment of photoreceptor status may be the most important clinical aspect for monitoring disease progression. Visual worsening in RP can also be due to cataract, cystoid macular edema, epiretinal membranes, macular holes and vitreomacular traction syndrome [5].

Optical coherence tomography (OCT) is a well-established method of analyzing in vivo retinal architecture [6] and has been used in the management of retinal diseases. Several OCT studies of RP have been reported, most of which show the capacity of OCT to recognize and follow retinal changes in RP patients, especially of the photoreceptors and their integrity, from a hyper-reflective zone in the outer retina, called as the ellipsoid zone (EZ) [5, 7–10]. The current study was undertaken to study and characterize the various morphological changes of photoreceptors on SD-OCT and to determine which of them are predictors for visual acuity loss in RP.

## Methods

This was a retrospective, observational, cross-sectional study of 224 eyes of 113 patients with RP, evaluated at a tertiary eye care center between 2011 and 2014 after approval from the Ethics Committee and Research Board of Narayana Nethralaya Eye Institute, Bangalore, India. The diagnosis of RP was based on a history of night vision problem, impairment in peripheral visual fields, reduction in electroretinogram rod and cone amplitudes using ISCEV (International Society for Clinical Electrophysiology of Vision) standard full-field electroretinograms [11] (in selected patients), and the presence of characteristic fundus changes. Only eyes with typical RP, defined clinically as the presence of disc pallor, arteriolar attenuation and bony spicules in the mid-peripheral fundus, with a clinically normal macula, with corrected distance visual acuity (CDVA) < 1.0 logMAR were included. Pedigree charts were included for the analysis whenever available.

Exclusion criteria were the presence of media opacities including cataract, an intraocular pressure of 21 mmHg or higher, inability to hold steady fixation, associated retinal abnormalities like cystoid macular edema, epi-retinal membrane, diabetic and other vitreomacular interface abnormalities, systemic conditions that could affect the visual system, history of ocular trauma, or concomitant ocular diseases, including a history of glaucoma, laser therapy, or ocular abnormalities affecting the cornea, lens, or optic nerve. Those eyes, where SD-OCT images were not available or were deemed not of good quality were also excluded.

**Fig. 1:**
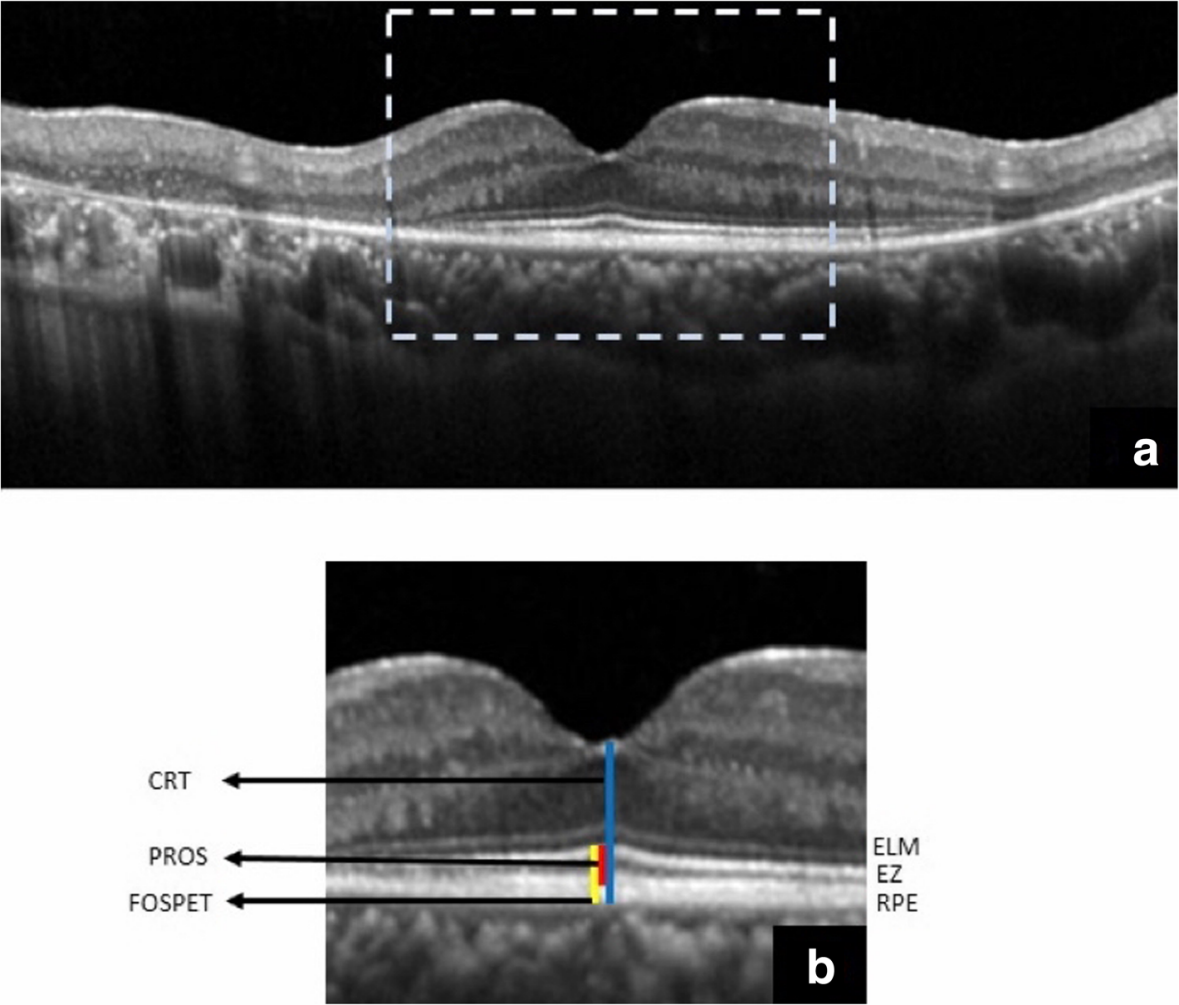
Magnification of the central OCT (dashed box in (**a**)) by 400% for measurement of the outer retinal features. ELM - external limiting membrane, EZ or IS/OS - ellipsoid zone, RPE retinal pigment epithelium, CRT - central retinal thickness, PROS - photoreceptor outer segment, FOSPET - foveal outer segment pigment epithelial thickness (**b**)

SD-OCT scans were acquired using the Spectral–domain Heidelberg retinal angiograph + optical coherence tomography (SD-HRA + OCT)© Heidelberg Engineering. The OCT component of the machine is a spectral/Fourier domain OCT with 5 μm axial resolution and an image acquisition speed of 40,000 scans/second. It employs a broad band 870 nm super luminescent diode source. Sixteen OCT scans were averaged to reduce noise. An experienced operator certified by the DARC (Digital Angiogram Reading Center, USA) acquired all images through a dilated pupil. Two independent observers (PBC and AKK) did the measurements in horizontal and vertical scans through the fovea by first magnifying the OCT scan 400% (Fig. 1a) and the values were averaged.

- The central retinal thickness (CRT): distance between the internal limiting membrane (ILM) and the outer border of the retinal pigment epithelium (RPE) at the fovea.
- The photoreceptor outer segment length (PROS) - distance between the inner border of the EZ and the inner border of RPE. [12]
- The foveal outer segment pigment epithelial thickness (FOSPET) - distance between the EZ and the outer border of the RPE, measured at the thinnest point of fovea (Fig. 1b). [13]

**Fig. 2:**
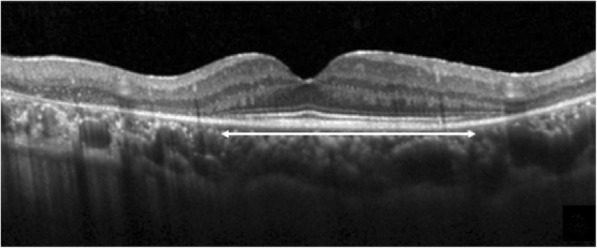
Measurement of the preserved ellipsoid zone length centered on the fovea

Extent of the preserved EZ line was also measured in horizontal and vertical scans, centered at the fovea (Fig. 2). All measurements were done using the calipers of the Heidelberg reader software [14].

To assess the inter-observer repeatability of the measurements of preserved EZ extent and measurements of CRT, PROS and FOSPET on SD-OCT images, the methods described by Bland and Altman were used [15]. The mean difference between two measurements (Observer1–Observer2) for each of the SD-OCT images represented the bias. The 95% limits of agreement (LoA), an expected difference between two measurements, were calculated as the mean of the differences ±1.96 standard deviation (SD) of the difference. The coefficient of repeatability (1.96 × SD of the difference), an indicator of the amount of variation that can be attributed to measurement error, was also calculated.

To ascertain the mean CDVA and across pedigree groups as well as for statistical analysis, Snellen’s visual acuity was converted to logMAR units and patients were categorized into 3 subgroups based on their visual acuity as follows:

1. Normal: CDVA = logMAR 0 in both eyes.
2. Mild visual impairment: logMAR 0 < CDVA < logMAR 0.477.
3. Moderate visual impairment: logMAR 0.477 < CDVA < logMAR 1. Data was analyzed by Shapiro Wilk test to test for normality. To determine the correlation between CDVA and the retinal microstructure on the SD-OCT, a linear mixed model (LMM) analysis was performed with CDVA as a dependent variable and SD-OCT values as covariates, as well as their interaction terms. All data were analyzed using the statistical software package SPSS version 22.0 (IBM Corporation, New York City, NY, USA).

## Results

### Patient data

Overall, 224 eyes of 113 patients were included in the study. These included both eyes of 111 patients and one eye in two patients. The other eye of these two patients were excluded due to a macular hole and post traumatic phthisical eye. 71 patients were male and 42 were females. Mean age of patients was 35.4 years (range 8–72 years). Mean CDVA was 0.33 logMAR (converted Snellen’s visual acuity = 20/42) with a range of 0.0–1.0 (SD = 0.25, Kurtosis = 0.38, skewness = 0.93).

On classifying the eyes based on the above mentioned criteria for vision, 54.5% (*n* = 122) had normal CDVA, 26.3% (*n* = 59) had mild visual impairment and 19.2% (*n* = 43) had moderate visual impairment. Pedigree charts were available for 72.5% (*n* = 82) patients. Most common inheritance pattern was the Simplex variant, seen in 34.5% (*n* = 39) followed by autosomal recessive (AR) (19.5%, *n* = 22), autosomal dominant (AD) (13.3%, *n* = 15) and X-linked (5.3%, *n* = 6). Mean CDVA across pedigree groups did not show a significant difference (Table 3).

### SD-OCT measurements

**Fig. 3:**
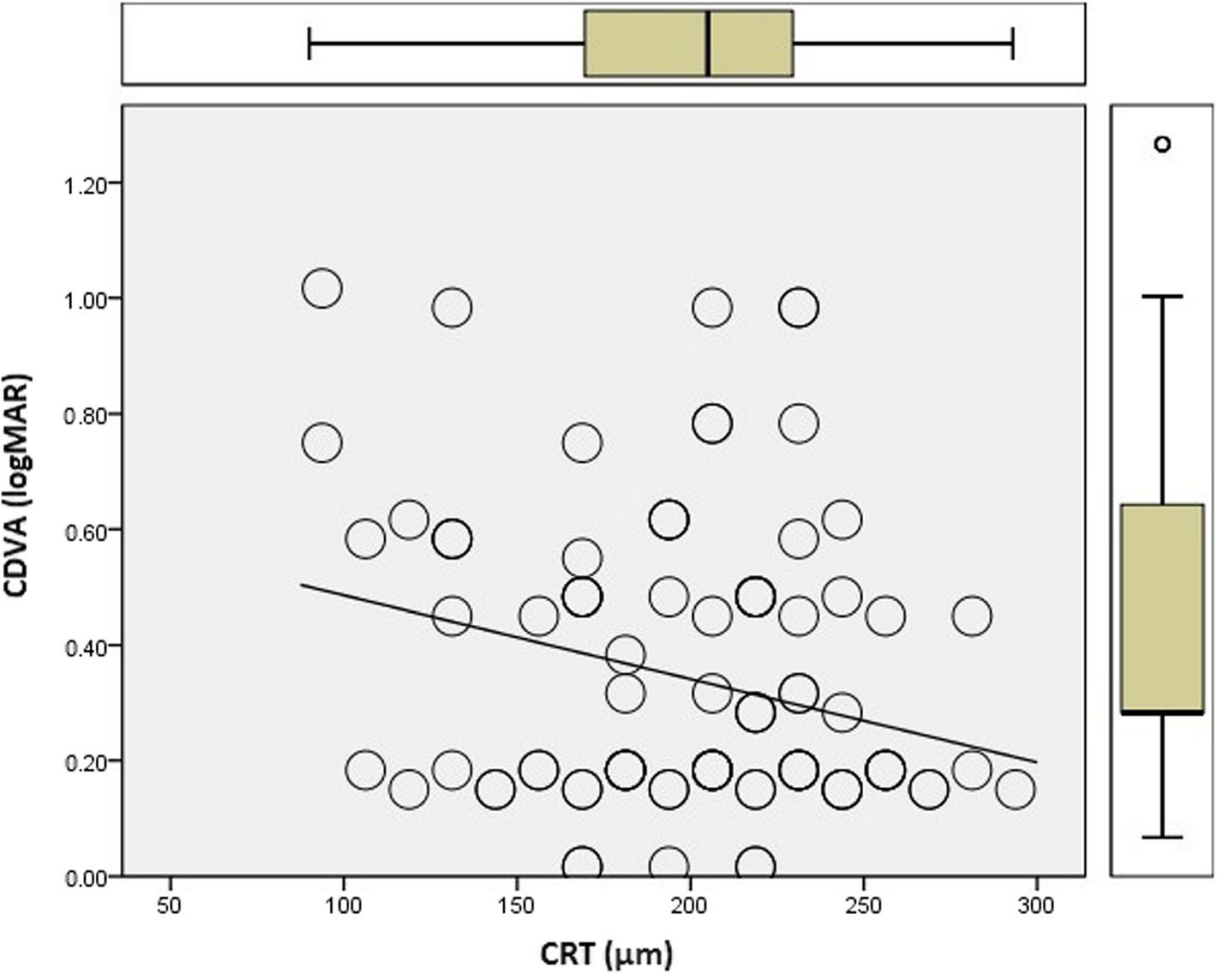
Scatter plot with central retinal thickness on the x-axis and CDVA on the y-axis

**Fig. 4:**
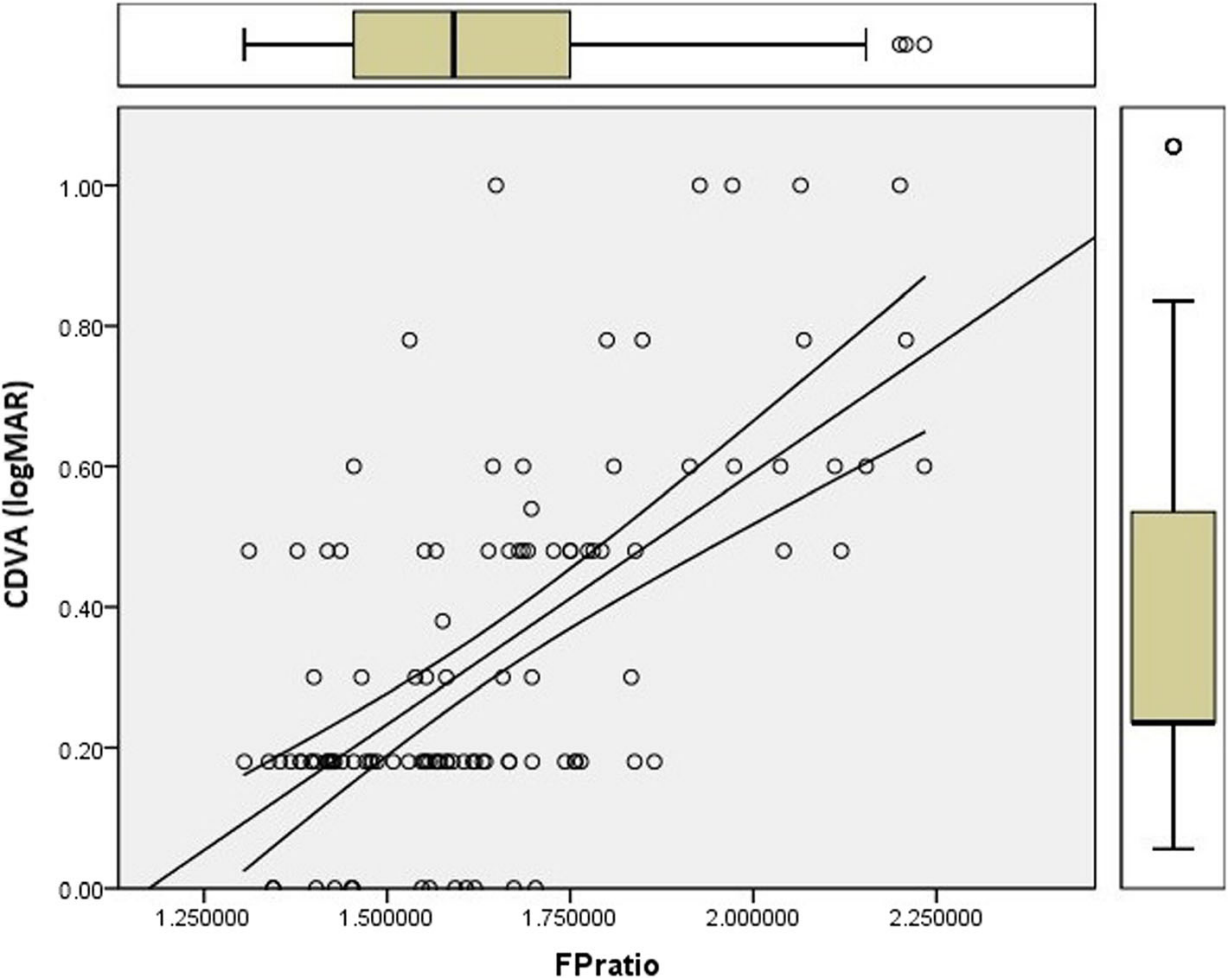
Scatter plot with the FP Ratio on the x-axis and CDVA on the y-axis

Mean values of SD-OCT measurements were CRT = 218.74 μm (range = 90–285 μm), PROS = 44.85 μm (range = 20–68 μm) and FOSPET = 71.15 μm (range = 45 – 95 μm). A new variable, termed the FOSPET-PROS ratio (FPR), was derived by dividing the FOSPET value by the PROS value. Mean FPR was 1.63 (range = 1.30–2.23). Mean values of CRT, PROS, FOSPET and FPR across visual acuity groups are given in Table 1 and show a significant difference. Multivariate analysis demonstrates that both CRT (*p* = 0.048) (Fig. 3) and FPR (*p* < 0.001) (Fig. 4) showed a significant correlation to logMAR CDVA; PROS and FOSPET however, did not show a significant correlation with CDVA as shown in Table 2. Age was adjusted for this analysis.

**Table 1:** Mean values of CRT, PROS, FOSPET and FPR across visual acuity groups

Visual acuity subgroup	Normal	Mild visual impairment	Moderate visual impairment	*P* value
CRT (μm)	237 ± 49	216 ± 40	173 ± 101	0.008
PROS (μm)	52 ± 9	38 ± 10	34 ± 9	0.001
FOSPET (μm)	79 ± 11	63 ± 12	61 ± 10	0.001
FPR	1.53 ± 0.13	1.65 ± 0.19	1.90 ± 0.23	< 0.001

*CRT* = central retinal thickness; *PROS* = photoreceptor outer segment; *FOSPET* = foveal outer segment pigment epithelial thickness; *FPR* = FOSPET:PROS ratio

**Table 2:** Age-adjusted multivariate analysis of CRT, PROS, FOSPET and FPR with CDVA as an independent variable

Measurement	Standardized coefficient	P value
CRT	−0.139	0.048
PROS	0.122	0.591
FOSPET	0.329	0.128
FPR	0.842	0.0001

*CRT* = central retinal thickness; *PROS* = photoreceptor outer segment; *FOSPET* = foveal outer segment pigment epithelial thickness; *FPR* = FOSPET:PROS ratio

Mean extent of the preserved EZ in the horizontal and vertical direction was 2856.4 μm (range = 518–8799 μm) and 2446.3 μm (range = 528–7914 μm), respectively, and strongly correlated to each other with r^2^ = 0.874, *p* < 0.001, but not to CDVA. None of these three measurements of the outer retina showed any significant correlation to preserved EZ extent.

Mean values of CDVA, CRT, PROS, FOSPET, FPR, and the horizontal and vertical extent of the preserved EZ in different pedigree subgroups are shown in Table 3. While the PROS showed a significant difference across pedigree groups (*p* = 0.44), none of the other variables were significantly different. The mean difference in CRT, PROS and FOSPET between Observer 1 and 2 were + 4 μm, + 3.5 μm and + 4.4 μm, respectively, and there was no significant bias between the observers (*p* = 0.953, *p* = 0.937, *p* = 0.924, respectively). The bias for the measurements of preserved EZ in the SD-OCT images was 10.4 μm and 11.2 μm in horizontal and vertical scan respectively with no significant bias between the observers (*p* = 0.983, *p* = 0.952, respectively).

**Table 3:** Mean values of CDVA and SD-OCT measurements in pedigree subgroups

Pedigree	CDVA (logMAR)	CRT (μm)	PROS (μm)	FOSPET (μm)	Horizontal EZ (μm)	Vertical EZ (μm)	FPR
Simplex	0.27 ± 0.20	211 ± 87	49 ± 11	75 ± 11	2803 ± 2137	2393 ± 1979	1.59 ± 0.21
AD	0.32 ± 0.19	227 ± 87	46 ± 12	72 ± 11	3147 ± 1988	2644 ± 2068	1.60 ± 0.22
AR	0.38 ± 0.22	232 ± 124	41 ± 13	66 ± 14	2926 ± 2254	2450 ± 1717	1.68 ± 0.24
X-linked	0.49 ± 0.30	222 ± 45	36 ± 12	64 ± 17	2897 ± 584	2880 ± 1316	1.82 ± 0.22
Not known	0.34 ± 0.25	216 ± 42	44 ± 13	71 ± 16	2722 ± 1778	2408 ± 1760	1.64 ± 0.20

*AD* = autosomal dominant; *AR* = autosomal recessive; *CDVA* = corrected distance visual acuity; *CRT* = central retinal thickness; *PROS* = photoreceptor outer segment; *FOSPET* = foveal outer segment pigment epithelial thickness; *EZ* = ellipsoid zone; *FPR* = FOSPET:PROS ratio

## Discussion

In recent years, several authors [16, 17] have reported on the utility of SD-OCT in the evaluation of eyes with RP, a disease that primarily affects the photoreceptors and the RPE [1]. In this study, the CRT, PROS length and FOSPET were measured. The extent of the preserved EZ at the fovea was measured as well. We also introduced a new variable obtained by dividing the FOSPET value by the PROS, called the FOSPET-PROS ratio (FPR).

The earliest histopathological change in the photoreceptors of eyes with RP is a shortening of the photoreceptor outer segments [18] while the loss of the cones is associated with reduced central vision at the end stage of the disease. Several OCT studies in RP patients study the correlation between the presence and continuity of the inner segment/outer segment (IS/OS) line, what is now called as EZ and the visual function [13, 17–24]. Using Fourier Domain OCT in eyes with retinal dystrophy, Lim et al. have demonstrated a 11% decrease in the macular inner retina layer and a 45% decrease in the macular ORL compared with normal [25]. Sandberg et al. [21] found a significant correlation between the grade of EZ and the visual acuity in RP patients. Aizawa et al. also reported that the length of the EZ significantly correlates with the retinal sensitivity and visual acuity of RP patients [24, 26]. The positive correlation between central visual acuity and extent of preserved EZ line possibly indicates that the degeneration of the central cones correlates with peripheral rods’ degeneration. In our study, we have found no significant correlation between CDVA, which signifies the cone function, and the extent of preserved EZ on SD-OCT. This could be attributed to early or late onset of cone degeneration secondary to the genetic heterogeneity of RP. Thus, the preserved EZ, rather than central visual acuity, may correlate better with visual field loss and retinal sensitivity on microperimetry.

Foveal thinning, probably secondary to photoreceptor loss, as measured on OCT has been shown to correlate with visual acuity in RP [13, 27]. In our study, the CRT significantly correlated with the CDVA. Photoreceptor loss has also been described using SD-OCT in other macular disorders [28, 29]. In RP patients, qualitative measures of photoreceptor structure such as external limiting membrane (ELM) status, foveal EZ and interdigitation zone (IZ) have been studied and have been shown to be significantly associated with central CDVA [8, 27, 30]. While PROS length has correlated with CDVA in many macular diseases [31–34], decreased PROS length is associated with loss of contrast sensitivity and color vision in RP patients with good CDVA [35]. Hence, we quantitatively measured the PROS length in our study. Foveal outer segment retinal epithelial thickness has been described as a quantitative measure of photoreceptor structure with correlation to the CDVA [13]. Neither PROS nor FOSPET significantly correlated with CDVA independently in our study. Additionally, we divided the value of FOSPET by the value of PROS to define a new variable called the FOSPET-PROS ratio or FPR. Using a multivariate model, we have demonstrated a significant inverse correlation between FPR and CDVA in RP. Moreover, the mean FPR was shown to be significantly different across various visual acuity groups. These correlations were independent of the age of the patient.

There are two possible explanations for the strong correlation of FPR with CDVA. The height of the RPE-Bruch’s complex may correlate with the height of photoreceptor outer segments, measured as PROS on SD-OCT. Instead of absolute values, a ratio may be a more accurate reflection of shortening of the photoreceptor outer segments, i.e. PROS shortening with reference to the RPE. Moreover, the RPE-Bruch’s complex is itself thickened and may reflect an additional pathology in the disease process, i.e. PROS thinning and RPE-Bruch’s complex thickening occur as the disease progresses despite a clinically normal appearing macula. Indeed, some histopathological reports mention the presence of acidic mucosal substances in the inter photoreceptor matrix space [36] and widespread deposits of abnormal material between the RPE and inner collagenous layer of Bruch’s membrane [37–39]. Although foveal thinning in RP is predominantly due to photoreceptor loss or shortening, on multivariate analysis the CRT independently correlated to the CDVA.

The CDVA, PROS, FOSPET and FPR showed a trend toward progressive decline when analyzed across pedigree subgroups with simplex or sporadic cases being the best and X-linked, the worst (Table 3); however, apart from PROS, the differences did not reach statistical significance. This further lends support to our hypothesis that the FPR, instead of absolute values of PROS, is a better surrogate marker for CDVA in RP patients.

Our study is an attempt to identify microstructural changes at the macula on SD-OCT that precede functional loss. Most patients of established RP have subnormal peripheral vision and heavily rely on central vision for their daily activities. Central visual acuity is dependent on photoreceptor survival at the macula. Identification of structural changes that precede visual loss is essential. To the best of our knowledge this is the first study that has quantitatively measured three vertical foveal parameters on OCT, the CRT, PROS and FOSPET, and their correlation with CDVA in RP patients. We have also measured the FOSPET: PROS ratio (FPR) and demonstrated that the FPR has the best correlation with CDVA compared to the other vertical foveal parameters and strongly believe that it is a potential marker of disease progression. Further research and establishment of a normative database of FPR might help us in early detection of progression of the disease, much before drop in visual acuity. Further, it might also help in monitoring treatment response. Studies are required to establish the role of the RPE-Bruch’s complex in patients with RP and the interaction of transverse outer retinal structures such as ELM, EZ and IZ with vertical measurements to clearly delineate the true measure and predictor of CDVA, contrast sensitivity and color vision in retinal degenerative disorders. It is possible that these SD-OCT measurements denote different aspects of photoreceptor functions. Identifying these markers can also help to formulate the inclusion and exclusion criteria for clinical trials in RP.

The limitations of our study are the use of Snellen’s visual acuity for measurement of CDVA, lack of documentation of transverse structural changes and their correlation to CDVA. We did not have the genetic mutational data for all patients and did not use other functional measures of central vision or a control group.

## Conclusion

There is a continuous need to detect micro structural changes in degenerative diseases like RP in its earliest stage when therapy may be most useful. To identify surrogate end points to monitor response to therapeutic intervention before true functional loss has occurred is the need of the hour.
